# Protein level identification of the *Listeria monocytogenes* Sigma H, Sigma L, and Sigma C regulons

**DOI:** 10.1186/1471-2180-13-156

**Published:** 2013-07-10

**Authors:** Sana Mujahid, Renato H Orsi, Kathryn J Boor, Martin Wiedmann

**Affiliations:** 1 Department of Food Science, Cornell University, 412 Stocking Hall, Ithaca, NY, USA

**Keywords:** *Listeria monocytogenes*, Alternative sigma factors, Quantitative proteomics

## Abstract

**Background:**

Transcriptional regulation by alternative sigma (σ) factors represents an important mechanism that allows bacteria to rapidly regulate transcript and protein levels in response to changing environmental conditions. While the role of the alternative σ factor σ^B^ has been comparatively well characterized in *L*. *monocytogenes*, our understanding of the roles of the three other *L*. *monocytogenes* alternative σ factors is still limited. In this study, we employed a quantitative proteomics approach using Isobaric Tags for Relative and Absolute Quantitation (iTRAQ) to characterize the *L*. *monocytogenes* σ^L^, σ^H^, and σ^C^ protein regulons. Proteomic comparisons used a quadruple alternative σ factor mutant strain (Δ*BCHL*) and strains expressing a single alternative σ factor (i.e., σ^L^, σ^H^, and σ^C^; strains Δ*BCH*, Δ*BCL*, and Δ*BHL*) to eliminate potential redundancies between σ factors.

**Results:**

Among the three alternative σ factors studied here, σ^H^ provides positive regulation for the largest number of proteins, consistent with previous transcriptomic studies, while σ^L^ appears to contribute to negative regulation of a number of proteins. σ^C^ was found to regulate a small number of proteins in *L*. *monocytogenes* grown to stationary phase at 37°C. Proteins identified as being regulated by multiple alternative σ factors include MptA, which is a component of a PTS system with a potential role in regulation of PrfA activity.

**Conclusions:**

This study provides initial insights into global regulation of protein production by the *L*. *monocytogenes* alternative σ factors σ^L^, σ^H^, and σ^C^. While, among these σ factors, σ^H^ appears to positively regulate the largest number of proteins, we also identified PTS systems that appear to be co-regulated by multiple alternative σ factors. Future studies should not only explore potential roles of alternative σ factors in activating a “cascade” of PTS systems that potentially regulate PrfA, but also may want to explore the σ^L^ and σ^C^ regulons under different environmental conditions to identify conditions where these σ factors may regulate larger numbers of proteins or genes.

## Background

The foodborne pathogen *Listeria monocytogenes* uses complex regulatory mechanisms to adapt to a variety of environmental conditions and to cause listeriosis, a life-threatening infection, in humans and animals. A key mechanism used by *L*. *monocytogenes* to regulate transcript and protein levels in order to adapt to changing environmental conditions is through alternative sigma (σ) factors. Alternative σ factors reprogram the RNA polymerase holoenzyme to recognize specific promoters and hence allow for rapid induction of transcription of potentially large groups of genes under specific environmental conditions [1]. In *L*. *monocytogenes*, four alternative σ factors, σ^B^, σ^C^, σ^H^, and σ^L^_,_ have been identified. However, σ^C^ has only been described in *L*. *monocytogenes* strains that group into lineage II, a well defined phylogenetic group that includes serotypes 1/2a and 1/2c [2-4]. A number of studies that have explored σ^B^-mediated stress response as well as σ^B^-mediated gene expression and protein production in *L*. *monocytogenes*[1,5-16] have shown that this alternative σ factor controls a large regulon and contributes to both stress response and virulence.

σ^H^, σ^L^, and σ^C^ have not been as extensively characterized as σ^B^ in *L*. *monocytogenes*, at least partially because studies to date have only identified limited phenotypic consequences of null mutations in these σ factors in *L*. *monocytogenes*. Among these three alternative σ factors, σ^H^ appears to control the largest regulon; Chaturongakul *et al*. (2011) identified 97 and 72 genes as positively and negatively regulated by σ^H^, respectively, in *L*. *monocytogenes* strain 10403S [7]. While a *L*. *monocytogenes* EGD-e *sigH* mutant was reported to have significantly impaired growth in minimal medium and under alkaline stress conditions as well as slightly reduced virulence potential in a mouse model [17], phenotypic studies in a *L*. *monocytogenes* 10403S Δ*sigH* strain did not find evidence for an effect of this mutation on virulence in a guinea pig model, cell invasion and intracellular growth, or resistance to heat stress [7]. With regard to σ^L^, 31 and 20 genes were identified as positively and negatively regulated, respectively, by this σ factor, in *L*. *monocytogenes* 10403S [7]. A more recent study in *L*. *monocytogenes* EGD-e identified 237 and 203 genes as positively regulated by σ^L^ when the parent and Δ*sigL* mutant strains were grown at 3°C and 37°C, respectively; most of the 47 genes that showed positive regulation by σ^L^ under both temperatures were located within prophage A118 [18]. Phenotypic and gene expression studies also support a potential contribution of σ^L^ to *L*. *monocytogenes* growth under different stress conditions, most notably osmotic and low temperature stress [19,20]. *L*. *monocytogenes* σ^L^ has also been reported to be involved in resistance to the antimicrobial peptide mesentericin Y105 [21]. Finally, studies conducted to date on the *L*. *monocytogenes* σ^C^ regulon typically identified few genes as σ^C^-dependent. Chaturongakul *et al*. (2011) were only able to identify and confirm, by qRT-PCR, a single gene (lmo0422) as σ^C^-dependent; lmo0422, which encodes LstR, a lineage II specific thermal regulator, is in the same operon as *sigC* and this finding is consistent with previous data suggesting that the *sigC* operon is auto-regulated [3,7]. Zhang *et al*. (2005) also found some evidence that σ^C^ may contribute to thermal resistance in the *L*. *monocytogenes* lineage II strain 10403S, when grown to log phase [3]; by contrast, Chaturongakul *et al*. (2011) did not find any evidence for reduced heat resistance when an independent *L*. *monocytogenes* 10403S Δ*sigC* strain was grown to stationary phase prior to heat exposure [7].

Previous studies [7] have suggested considerable overlap between different *L*. *monocytogenes* alternative σ factor regulons (e.g., between the σ^B^ and the σ^H^ regulon), suggesting the potential for redundancies as well as compensation for deletion of a single alternative σ factor by other σ factors. We thus hypothesized that an experimental approach that eliminates these potential redundancies is needed to gain a better understanding of the roles of σ^C^, σ^H^, and σ^L^ in regulating production of specific proteins in *L*. *monocytogenes*. As an experimental approach, we selected to create an *L*. *monocytogenes* 10403S quadruple mutant with a non-polar deletion of all four genes that encode alternative σ factors (i.e., strain Δ*BCHL*) as well as corresponding mutants with deletions of three alternative σ factors (Δ*BCH*, Δ*BCL*, and Δ*BHL*), which thus expressed only σ^L^, σ^H^, and σ^C^, respectively. These strains were then used for proteomic comparisons between the quadruple mutant strain and the three different strains expressing only a single alternative σ factor. We particularly focused on exploring the contributions of these alternative σ factors to regulating protein production as, despite availability of a number of proteomics data sets on the σ^B^ regulon [15,16], only a single proteomics study on the σ^L^ regulon is available [22]. While alternative σ factors directly regulate transcription of genes, it is increasingly clear that alternative σ factors also make important indirect contributions to protein production via mechanisms other than transcriptional activation of a σ factor dependent promoter upstream of a protein encoding gene, including through regulation of non-coding RNAs or through direct transcriptional up-regulation of a protein that in turn, directly or indirectly, affects production of other proteins [23]. The goal of this proteomics study was thus to specifically identify additional post-transcriptional regulatory pathways that are linked to the action of alternative σ factors in *L*. *monocytogenes*.

## Results and discussion

Proteomic comparisons between *L*. *monocytogenes* mutants expressing only σ^L^, σ^H^, and σ^C^ and a quadruple mutant that does not express any alternative σ factors, all grown to stationary phase at 37°C, showed that (i) σ^H^ provides, among these three alternative σ factors, positive regulation for the largest number of proteins, consistent with previous transcriptomic studies [7]; (ii) σ^L^ appears to contribute to negative regulation of a number of proteins; (iii) σ^C^ regulates a small number of proteins in *L*. *monocytogenes* grown to stationary phase at 37°C; and (iv) proteins regulated by multiple alternative σ factors include MptA, which has a potential role in regulation of PrfA.

### σ^H^ positively regulates a large number of proteins and appears to directly and indirectly contribute to transport and metabolism of β-glucosides

**Figure 1:**
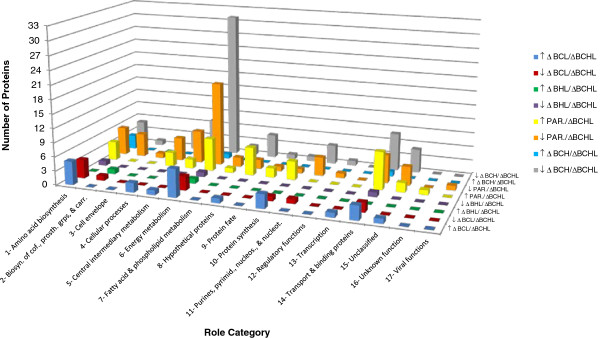
**Functional role category classification of alternative σ factor dependent proteins.** Functional role category classification of σ^H^ positively-regulated (blue), σ^H^ negatively-regulated (red), σ^C^ positively-regulated (green), σ^C^ negatively-regulated (purple), σ^L^ positively-regulated (turquoise), and σ^L^ negatively-regulated (gray) proteins; and proteins with higher levels in *L*. *monocytogenes* parent strain 10403S (PAR.) compared to Δ*BCHL* (yellow) and lower levels in PAR. compared to Δ*BCHL* (orange). Role category numbers correspond to: (1) Amino acid biosynthesis; (2) Biosynthesis of cofactors, prosthetic groups, and carriers; (3) Cell envelope; (4) Cellular processes; (5) Central intermediary metabolism; (6) Energy metabolism; (7) Fatty acid and phospholipid metabolism; (8) Hypothetical proteins; (9) Protein fate; (10) Protein synthesis; (11) Purines, pyrimidines, nucleosides, and nucleotides; (12) Regulatory functions; (13) Transcription; (14) Transport and binding proteins; (15) Unclassified; (16) Unknown function; (17) Viral functions. One protein may be classified into more than one role category. Statistical analysis of contingency tables for regulons with > 10 proteins (i.e., proteins positively regulated by σ^H^; proteins negatively regulated by σ^L^; proteins with higher or lower levels in the parent strain) found that role categories were not randomly distributed among proteins negatively regulated by σ^L^ and proteins with lower levels in the parent strain.

Our proteomic comparison identified 15 proteins as positively regulated by σ^H^, as supported by higher protein levels (Fold change (FC) ≥ 1.5; *p*-value^c^ (*p*^c^) < 0.05) in *L*. *monocytogenes* Δ*BCL* as compared to the Δ*BCHL* strain (Table 1); four of these 15 proteins also showed higher levels in the parent strain (which expresses all four alternative σ factors) as compared to the quadruple mutant. Overall, positive fold changes for these proteins (in Δ*BCL* versus Δ*BCHL*) ranged from 1.55 to 3.39. These 15 proteins represented nine role categories (e.g., “energy metabolism”; “amino acid biosynthesis”; “transport and binding proteins”, see Figure 1); a Monte Carlo simulation of Fisher’s exact test did not find a significant association between positively regulated genes and role categories (*p* = 0.06); however, individual Fisher’s exact tests did show overrepresentation of proteins in the role category “amino acid biosynthesis” among the 15 proteins that were found to be positively regulated by σ^H^ (with a significant *p*-value; *p* < 0.01; Odds Ratio = 6.26). Some of the 15 proteins positively regulated by σ^H^ have likely roles in stress adaptation and virulence, including Lmo1439 (superoxide dismutase, SodA) [24] and Lmo0096 (mannose-specific PTS system IIAB component, MptA), which has been linked to regulation of the virulence gene regulator PrfA [25]. Previously reported transcriptomic studies [7] only identified the coding gene for one of these 15 proteins (i.e., Lmo1454) as σ^H^-dependent; lmo1454 (*rpoD*) was also identified as preceded by a σ^H^ consensus promoter, suggesting direct transcriptional regulation by σ^H^. In addition, the coding gene for Lmo2487, one of these 15 proteins, is in an operon with lmo2485, which was previously reported to be positively regulated by σ^H^, even though no upstream σ^H^ consensus promoter was identified, suggesting indirect regulation [7]. RNA-Seq data from our group (unpublished data) found clear evidence (FC > 5 and likelihood of being positively regulated by σ^H^ > 0.95) for σ^H^-dependent transcript levels for only two of the genes encoding these 15 proteins, including lmo1454 and lmo0239; importantly, RNA-Seq data allow for quantification with similar sensitivity as qRT-PCR [14]. lmo1454 thus has been consistently identified as a gene that is directly up-regulated by σ^H^, as supported by proteomics and transcriptomic studies and identification of an upstream σ^H^-dependent promoter. Many of the other proteins identified here as showing σ^H^-dependent production, on the other hand, appear to be regulated indirectly by σ^H^, possibly at the post-transcriptional level. While future efforts will be needed to confirm σ^H^-dependent production of these proteins (e.g., through Western blot or translational reporter fusions) and to explore the mechanisms of regulation, our data identified and further characterized a σ^H^-dependent pathway that involves indirect effects of σ^H^. Specifically, we found that both Lmo0027 (a component of a β-glucoside specific PTS system) and BglA (a β-glucosidase) showed higher protein levels in the presence of σ^H^. As lmo0027 is preceded by a σ^H^ consensus promoter, these findings suggest a model where σ^H^ directly activates transcription of lmo0027, which facilitates PTS-based import of beta-glucosides into the cell. We hypothesize that these β-glucosides then lead to an increase in the levels of BglA (through a yet to be defined mechanism), facilitating the use of β-glucosides in downstream pathways involved in energy acquisition (e.g., glycolysis, the pentose phosphate pathway).

**Table 1:** **Proteins found to be differentially regulated by** σ^**H**^, **as determined by a proteomic comparison between *L***. ***monocytogenes *10403S Δ*BCL *and Δ*BCHL***

**Protein**^**a**^	**Fold change Δ *BCL */Δ*BCHL***	**Description**	**Gene name**	**Role category**^**b**^	**Sub**-**Role category**^**b**^	**Promoter**^**d**^	**Sigma factor**
**Proteins with positive fold change (>1**.**5) and *p *< 0**.**05 (indicating positive regulation by σ**^**H**^**)**							
Lmo0027	1.55	beta-glucoside-specificPTS system IIABC component	*lmo0027*	Transport and binding proteins	Carbohydrates, organic alcohols, and acids	aggacacgtgtatgcgtggagtcctcgaatga	SigmaH
				Amino acid biosynthesis	Aromatic amino acid family		
				Energy metabolism	Pyruvate dehydrogenase		
Lmo0096	3.39	mannose-specific PTS system IIAB component ManL	*mptA*	Energy metabolism	Pyruvate dehydrogenase	tggcacagaacttgca	SigmaL
				Amino acid biosynthesis	Aromatic amino acid family		
				Transport and binding proteins	Carbohydrates, organic alcohols, and acids		
Lmo0239	1.82	cysteinyl-tRNA synthetase	*cysS*	Protein synthesis	tRNA aminoacylation	ttgcaaggaattttattgctgttataatag	SigmaA
Lmo0319	1.77	beta-glucosidase	*bglA*	Energy metabolism	Sugars	N/A	N/A
Lmo0356	2.16	YhhX family oxidoreductase	*lmo0356*	Energy metabolism	Fermentation	tggctaagtacagcgctagtgtagtactat	SigmaA
				Energy metabolism	Electron transport		
				Central intermediary metabolism	Other		
Lmo1001	1.65	hypothetical protein	*lmo1001*	Unclassified	Role category not yet assigned	N/A	N/A
Lmo1070	2.18	similar to B. subtilis YlaN protein	*lmo1070*	Hypothetical proteins	Conserved	ttgcgtggcaaataaattatgctatact	SigmaA
Lmo1255	1.60	trehalose-specific PTS system IIBC component	*lmo1255*	Energy metabolism	Pyruvate dehydrogenase	ttgcgctttcaactgatttatagtatagt	SigmaA
				Amino acid biosynthesis	Aromatic amino acid family		
				Transport and binding proteins	Carbohydrates, organic alcohols, and acids		
Lmo1439	1.66	superoxide dismutase	*sodA*	Cellular processes	Detoxification	ttgcaagcatttagggagcatggtaggct	SigmaA
						gtttaacttttgagtttcagggaaa	SigmaB
Lmo1454^c^	1.85	RNA polymerase sigma factor RpoD	*rpoD*	Transcription	Transcription factors	gttttaaaaccgctaaatgatggtat	SigmaB
						aggacttttgctttttgtggcgaatat	SigmaH
						ttgactttttagcaaaaatacagtatctt	SigmaA
Lmo2006	1.60	acetolactate synthase catabolic	*alsS*	Amino acid biosynthesis	Aspartate family	ttgcaataattcttttgagtagtataat	SigmaA
				Amino acid biosynthesis	Pyruvate family		
Lmo2064	2.01	large conductance mechanosensitive channel protein	*mscL*	Cellular processes	Adaptations to atypical conditions	tttcacatcgcagttagatgttttatact	SigmaA
Lmo2487	1.65	hypothetical protein	*lmo2487*	Hypothetical proteins	Conserved	N/A	N/A
Lmo2614	2.05	50S ribosomal protein L30	*rpmD*	Protein synthesis	Ribosomal proteins: synthesis and modification	ttgattactacccctaacccgtgtataat	SigmaA
Lmo2621	1.63	50S ribosomal protein L24	*rplX*	Protein synthesis	Ribosomal proteins: synthesis and modification	ttgattactacccctaacccgtgtataat	SigmaA
**Proteins with negative fold change (<** -**1**.**5) and *p *< 0**.**05 (indicating negative regulation by σ**^**H**^**)**							
Lmo1877	−1.61	formate-tetrahydrofolate ligase	*fhs*	Amino acid biosynthesis	Aspartate family		
				Protein synthesis	tRNA aminoacylation		
				Amino acid biosynthesis	Histidine family		
				Purines, pyrimidines, nucleosides, and nucleotides	Purine ribonucleotide biosynthesis		
				Biosynthesis of cofactors, prosthetic groups, and carriers	Pantothenate and coenzyme A		
Lmo2094	−7.35	hypothetical protein	*lmo2094*	Energy metabolism	Sugars		
Lmo2097	−3.17	galactitol-specific PTS system IIB component	*lmo2097*	Energy metabolism	Pyruvate dehydrogenase		
				Amino acid biosynthesis	Aromatic amino acid family		
				Transport and binding proteins	Carbohydrates, organic alcohols, and acids		
Lmo2098	−2.33	galactitol-specific PTS system IIA component	*lmo2098*	Energy metabolism	Pyruvate dehydrogenase		
				Amino acid biosynthesis	Aromatic amino acid family		
				Transport and binding proteins	Carbohydrates, organic alcohols, and acids		

^a^Protein names are based on the *L. monocytogenes* EGD-e locus.

^b^Role Categories and Sub-Role categories are based on JCVI classification [26].

^c^Reported as positively and directly regulated by σ^H^ in Chaturongakul *et al*., 2011 [7].

^d^Promoters were identified based on RNA-Seq data (Orsi *et al*., unpublished) or previously published data. -10 and -35 (σ^A^, σ^B^, σ^H^) and -12 and -24 (σ^L^) regions are underlined. N/A indicates that a promoter was not identified.

Our proteomic comparison also identified four proteins that showed lower levels in the strain expressing σ^H^, suggesting (indirect) negative regulation by σ^H^; three of these four proteins also showed lower levels in the parent strain (which expresses all four alternative σ factors) as compared to the quadruple mutant. None of the genes encoding these proteins showed significantly higher transcript levels in a Δ*sigH* strain in a transcriptomic study [7]. However, the coding gene for Lmo1877, one of these four proteins, is in an operon with lmo1876, which was previously reported to be negatively regulated by σ^H^[7]. Overall, global indirect down-regulation of proteins by σ^H^ does not seem to play an important role in stationary phase *L*. *monocytogenes* 10403S.

### σ^L^ appears to contribute to negative regulation of a number of proteins

Our proteomic comparison identified only two proteins (Lmo0096 and Lmo2006) as positively regulated by σ^L^, as supported by higher protein levels (FC ≥ 1.5; *p*^c^ < 0.05) in *L*. *monocytogenes* Δ*BCH* as compared to the Δ*BCHL* strain (Table 2). Both of these proteins also showed higher levels in the parent strain (which expresses all four alternative σ factors) as compared to the quadruple mutant. Lmo0096 (MptA) is annotated as the mannose-specific PTS system IIAB component, while Lmo2006 (AlsS) is annotated as an acetolactate synthase. Both lmo0096 and lmo2006 have previously been reported to be positively regulated by σ^L^ at the transcriptomic level [18]. Lmo0096 was also reported as showing lower levels in an *L*. *monocytogenes* EGD-e *rpoN* (σ^L^) mutant in a 2-DE based proteomic analysis [22] and the lmo0096 gene was found to be preceded by a putative σ^L^ consensus promoter in the same study, further supporting positive regulation of the gene encoding this protein by σ^L^.

**Table 2:** **Proteins found to be differentially regulated by σ**^**L**^, **as determined by a proteomic comparison between *L***. ***monocytogenes *10403S Δ*BCH *and Δ*BCHL***

**Protein**^**a**^	**Fold change Δ *BCH */Δ*BCHL***	**Description**	**Gene name**	**Role category**^**b**^	**Sub**-**Role category**^**b**^
**Proteins with positive fold change (>1**.**5) and *p *< 0**.**05 (indicating positive regulation by σ**^**L**^**)**					
Lmo0096^d,f^	64.16	mannose-specific PTS system IIAB component ManL	*mptA*	Energy metabolism	Pyruvate dehydrogenase
				Amino acid biosynthesis	Aromatic amino acid family
				Transport and binding proteins	Carbohydrates, organic alcohols, and acids
Lmo2006^g^	3.41	acetolactate synthase catabolic	*alsS*	Amino acid biosynthesis	Aspartate family
				Amino acid biosynthesis	Pyruvate family
**Proteins with negative fold change (<** -**1**.**5) and *p *< 0**.**05 (indicating negative regulation by σ**^**L**^**)**					
Lmo0027^c,e^	−3.62	beta-glucoside-specific PTS system IIABC component	*lmo0027*	Transport and binding proteins	Carbohydrates, organic alcohols, and acids
				Amino acid biosynthesis	Aromatic amino acid family
				Energy metabolism	Pyruvate dehydrogenase
Lmo0130	−3.64	hypothetical protein	*lmo0130*	Unclassified	Role category not yet assigned
Lmo0178	−2.07	hypothetical protein	*lmo0178*	Regulatory functions	Other
Lmo0181	−3.25	multiple sugar transport system substrate-binding protein	*lmo0181*	Transport and binding proteins	Unknown substrate
Lmo0260	−1.68	hydrolase	*lmo0260*	Hypothetical proteins	Conserved
Lmo0278	−1.67	maltose/maltodextrin transport system ATP-binding protein	*lmo0278*	Transport and binding proteins	Carbohydrates, organic alcohols, and acids
Lmo0319^c,e^	−2.96	beta-glucosidase	*bglA*	Energy metabolism	Sugars
Lmo0343	−3.94	transaldolase	*tal2*	Energy metabolism	Pentose phosphate pathway
Lmo0344	−4.69	short chain dehydrogenase	*lmo0344*	Energy metabolism	Biosynthesis and degradation of polysaccharides
Lmo0345	−6.04	ribose 5-phosphate isomerase B	*lmo0345*	Energy metabolism	Pentose phosphate pathway
Lmo0346	−2.74	triosephosphate isomerase	*tpiA2*	Energy metabolism	Glycolysis/gluconeogenesis
Lmo0348	−2.41	dihydroxyacetone kinase	*lmo0348*	Fatty acid and phospholipid metabolism	Biosynthesis
				Energy metabolism	Sugars
Lmo0391	−1.67	hypothetical protein	*lmo0391*		
Lmo0401	−2.16	alpha-mannosidase	*lmo0401*	Unclassified	Role category not yet assigned
Lmo0517^e^	−3.21	phosphoglycerate mutase	*lmo0517*	Energy metabolism	Glycolysis/gluconeogenesis
Lmo0521	−2.23	6-phospho-beta-glucosidase	*lmo0521*	Energy metabolism	Sugars
Lmo0536	−1.97	6-phospho-beta-glucosidase	*lmo0536*	Central intermediary metabolism	Other
Lmo0574	−1.65	6-phospho-beta-glucosidase GmuD	*lmo0574*	Central intermediary metabolism	Other
Lmo0640	−1.78	oxidoreductase	*lmo0640*	Energy metabolism	Fermentation
				Central intermediary metabolism	Other
				Energy metabolism	Electron transport
Lmo0643	−2.61	transaldolase	*lmo0643*	Energy metabolism	Pentose phosphate pathway
Lmo0689	−1.71	chemotaxis protein CheV	*lmo0689*	Cellular processes	Chemotaxis and motility
Lmo0690	−2.44	flagellin	*flaA*	Cellular processes	Chemotaxis and motility
Lmo0692	−1.66	chemotaxis protein CheA	*cheA*	Cellular processes	Chemotaxis and motility
Lmo0813	−2.04	fructokinase	*lmo0813*	Energy metabolism	Sugars
Lmo0930	−1.88	hypothetical protein	*lmo0930*	Unclassified	Role category not yet assigned
Lmo1242	−1.59	hypothetical protein	*lmo1242*	Hypothetical proteins	Conserved
Lmo1254	−2.10	alpha-phosphotrehalase	*lmo1254*	Energy metabolism	Biosynthesis and degradation of polysaccharides
Lmo1348	−2.42	glycine cleavage system T protein	*gcvT*	Energy metabolism	Amino acids and amines
Lmo1349	−2.68	glycine cleavage system P-protein	*gcvPA*	Energy metabolism	Amino acids and amines
				Central intermediary metabolism	Other
Lmo1350^e^	−2.11	glycine dehydrogenase subunit 2	*gcvPB*	Central intermediary metabolism	Other
				Energy metabolism	Amino acids and amines
Lmo1388^e^	−2.02	ABC transport system	*tcsA*	Unclassified	Role category not yet assigned
Lmo1389	−2.32	simple sugar transport system ATP-binding protein	*lmo1389*	Transport and binding proteins	Carbohydrates, organic alcohols, and acids
Lmo1538^e^	−1.89	glycerol kinase	*glpK*	Energy metabolism	Other
Lmo1699	−1.92	Methyl-accepting chemotaxis protein	*lmo1699*	Cellular processes	Chemotaxis and motility
Lmo1730	−2.55	lactose/L-arabinose transport system substrate-binding protein	*lmo1730*	Transport and binding proteins	Carbohydrates, organic alcohols, and acids
Lmo1791	−1.75	hypothetical protein	*lmo1791*		
Lmo1812	−1.70	L-serine dehydratase iron-sulfur-dependent alpha subunit	*lmo1812*	Energy metabolism	Amino acids and amines
				Energy metabolism	Glycolysis/gluconeogenesis
Lmo1856	−1.65	purine nucleoside phosphorylase	*deoD*	Purines, pyrimidines, nucleosides, and nucleotides	Salvage of nucleosides and nucleotides
Lmo1860	−1.64	peptide-methionine (S)-S-oxide reductase	*msrA*	Protein fate	Protein modification and repair
Lmo1877	−2.14	formate-tetrahydrofolate ligase	*fhs*	Amino acid biosynthesis	Aspartate family
				Protein synthesis	tRNA aminoacylation
				Amino acid biosynthesis	Histidine family
				Purines, pyrimidines, nucleosides, and nucleotides	Purine ribonucleotide biosynthesis
				Biosynthesis of cofactors, prosthetic groups, and carriers	Pantothenate and coenzyme A
Lmo1954^e^	−1.97	phosphopentomutase	*deoB*	Purines, pyrimidines, nucleosides, and nucleotides	Salvage of nucleosides and nucleotides
Lmo1993	−1.81	pyrimidine-nucleoside phosphorylase	*pdp*	Purines, pyrimidines, nucleosides, and nucleotides	Salvage of nucleosides and nucleotides
Lmo2094	−28.99	hypothetical protein	*lmo2094*	Energy metabolism	Sugars
Lmo2097	−12.12	galactitol-specific PTS system IIB component	*lmo2097*	Energy metabolism	Pyruvate dehydrogenase
				Amino acid biosynthesis	Aromatic amino acid family
				Transport and binding proteins	Carbohydrates, organic alcohols, and acids
Lmo2098	−3.96	galactitol-specific PTS system IIA component	*lmo2098*	Energy metabolism	Pyruvate dehydrogenase
				Amino acid biosynthesis	Aromatic amino acid family
				Transport and binding proteins	Carbohydrates, organic alcohols, and acids
Lmo2160	−2.37	sugar phosphate isomerase/epimerase	*lmo2160*	Hypothetical proteins	Conserved
Lmo2161	−2.58	hypothetical protein	*lmo2161*	Hypothetical proteins	Conserved
Lmo2362	−1.87	glutamate/gamma-aminobutyrate antiporter	*lmo2362*	Transport and binding proteins	Amino acids, peptides and amines
Lmo2425	−1.59	glycine cleavage system H protein	*gcvH*	Energy metabolism	Amino acids and amines
Lmo2481	−1.52	pyrophosphatase PpaX	*ppaX*	Central intermediary metabolism	Other
Lmo2529	−1.72	ATP synthase F1 beta subunit	*atpD2*	Energy metabolism	ATP-proton motive force interconversion
Lmo2648	−2.50	hypothetical protein	*lmo2648*	Unclassified	Role category not yet assigned
Lmo2664	−1.72	L-iditol 2-dehydrogenase	*lmo2664*	Central intermediary metabolism	Other
				Energy metabolism	Glycolysis/gluconeogenesis
				Energy metabolism	Electron transport
				Energy metabolism	TCA cycle
				Energy metabolism	Fermentation
Lmo2696	−2.68	dihydroxyacetone kinase L subunit	*lmo2696*	Energy metabolism	Sugars
				Fatty acid and phospholipid metabolism	Biosynthesis
Lmo2697	−3.10	dihydroxyacetone kinase	*lmo2697*	Hypothetical proteins	Conserved
Lmo2743	−2.71	transaldolase	*tal1*	Energy metabolism	Pentose phosphate pathway

^a^Protein names are based on the *L. monocytogenes* EGD-e locus.

^b^Role Categories and Sub-Role categories are based on JCVI classification [26].

^c^Reported as negatively regulated by σ^L^ in Chaturongakul *et al*., 2011 [7].

^d^Reported as downregulated in a *rpoN* (σ^L^) mutant compared to wildtype *L. monocytogenes* EGD-e in Arous *et al*., 2004 [22].

^e^Reported as upregulated in a *rpoN* (σ^L^) mutant compared to wildtype *L. monocytogenes* EGD-e in Arous *et al*., 2004 [22].

^f^Preceded by a putative σ^L^ promoter; tggcacagaacttgca; -12 and -24 regions are underlined.

^g^Preceded by a putative σ^A^ promoter; ttgcaataattcttttgagtagtataat; -10 and -35 regions are underlined.

A total of 56 proteins showed lower levels in the presence of σ^L^ (in the comparison between the Δ*BCH* and the Δ*BCHL* strain), suggesting indirect negative regulation of these proteins by σ^L^ (Table 2); two of the genes encoding these proteins had previously been shown to have higher transcript levels in a Δ*sigL* null mutant as compared to a parent strain, further supporting negative regulation by σ^L^[7]. Twenty-one of the proteins with evidence for negative regulation by σ^L^ also showed lower protein levels in the parent strain as compared to the Δ*BCHL* strain (Additional file 1: Table S1), further supporting their negative regulation. Four of these 21 proteins as well as three other proteins found to be negatively regulated by σ^L^ in this study were also reported as showing higher transcript levels in an *L*. *monocytogenes* EGD-e *rpoN* (σ^L^) mutant [22] (Table 2), supporting their negative regulation by σ^L^. Overall, the 56 proteins identified here as negatively regulated by σ^L^ represented 13 role categories (e.g., energy metabolism, transport and binding proteins, central intermediary metabolism), including 31 proteins in the energy metabolism role category; statistical analyses showed overrepresentation of the role category “energy metabolism” (*p* < 0.01; Odds Ratio = 5.6) among these 56 proteins. Specific proteins identified as negatively regulated by σ^L^ included flagellin (FlaA), chemotaxis protein CheA, and a glutamate-γ-aminobutyric acid (GABA) antiporter (Lmo2362, GadC, GadT2), which have known roles in stress adaptation or virulence in *L*. *monocytogenes*[1,27].

### σ^C^ regulates a small number of proteins

Previous studies indicated a role for σ^C^ in *L*. *monocytogenes* thermal adaptive response as well as in cold adaptation [3,13], however only a few genes have been identified as part of the σ^C^ regulon [7]. Similarly, we were only able to identify one protein, Lmo0096, that showed higher protein levels (FC ≥ 1.5; *p*^c^ < 0.05) in the presence of σ^C^ (i.e., the comparison between the Δ*BHL* and the Δ*BCHL* strain; Table 3). Lmo0096 has been previously reported to be induced under cold stress in *L*. *monocytogenes*[28], supporting a role of σ^C^ in response to temperature stress in the bacterium. By comparison, the transcriptomic study by Chaturongakul *et al*., 2011 only identified lmo0422, which is in the same operon as *sigC* (lmo0423), as positively regulated by σ^C^[7].

**Table 3:** **Proteins found to be differentially regulated by σ**^**C**^, **as determined by a proteomic comparison between *L***. ***monocytogenes *10403S Δ*BHL *and Δ*BCHL***

**Protein**^**a**^	**Fold change Δ*BHL***/**Δ*BCHL***	**Description**	**Gene name**	**Role category**^**b**^	**Sub**-**Role category**^**b**^
**Proteins with positive fold change (>1**.**5) and *p *< 0**.**05 (indicating positive regulation by σ**^**C**^**)**					
Lmo0096^c^	3.19	mannose-specific PTS system IIAB component ManL	*mptA*	Energy metabolism	Pyruvate dehydrogenase
				Amino acid biosynthesis	Aromatic amino acid family
				Transport and binding proteins	Carbohydrates, organic alcohols, and acids
**Proteins with negative fold change (<** -**1**.**5) and *p *< 0**.**05 (indicating negative regulation by σ**^**C**^**)**					
Lmo2094	−1.82	hypothetical protein	*lmo2094*	Energy metabolism	Sugars
Lmo1902	−1.61	3-methyl-2-oxobutanoate hydroxymethyltransferase	*panB*	Biosynthesis of cofactors, prosthetic groups, and carriers	Pantothenate and coenzyme A

^a^Protein names are based on the *L. monocytogenes* EGD-e locus.

^b^Role Categories and Sub-Role categories are based on JCVI classification [26].

^c^Preceded by a putative σ^L^ promoter; tggcacagaacttgca; -12 and -24 regions are underlined.

We also identified two proteins, Lmo2094 and Lmo1902, that showed higher protein levels in the absence of σ^C^, suggesting negative regulation of these proteins by σ^C^ (Table 3). By comparison, the transcriptomic study by Chaturongakul *et al*. (2011) identified three different genes, representing two operons (lmo1854; lmo2185 and lmo2186), that showed lower transcript levels in the parent strain compared to the Δs*igC* mutant, suggesting negative regulation by σ^C^[7]. While our data are consistent with previous findings of a limited σ^C^ regulon in *L*. *monocytogenes* 10403S, it is possible that σ^C^- dependent gene regulation only occurs under specific conditions (e.g., heat stress [3]) and that more complete identification of the σ^C^ regulon requires transcriptomic and proteomic studies under specific conditions that remain to be defined. In addition, future experiments using an *L*. *monocytogenes* strain that expresses *sigC* from an inducible promoter may also allow for identification of additional proteins that show σ^C^-dependent production; this strategy applied to other alternative σ factors may also allow for identification of additional proteins that show σ^H^- or σ^L^-dependent production.

### Proteins regulated by multiple alternative σ factors include MptA, which has a potential role in regulation of PrfA

**Figure 2:**
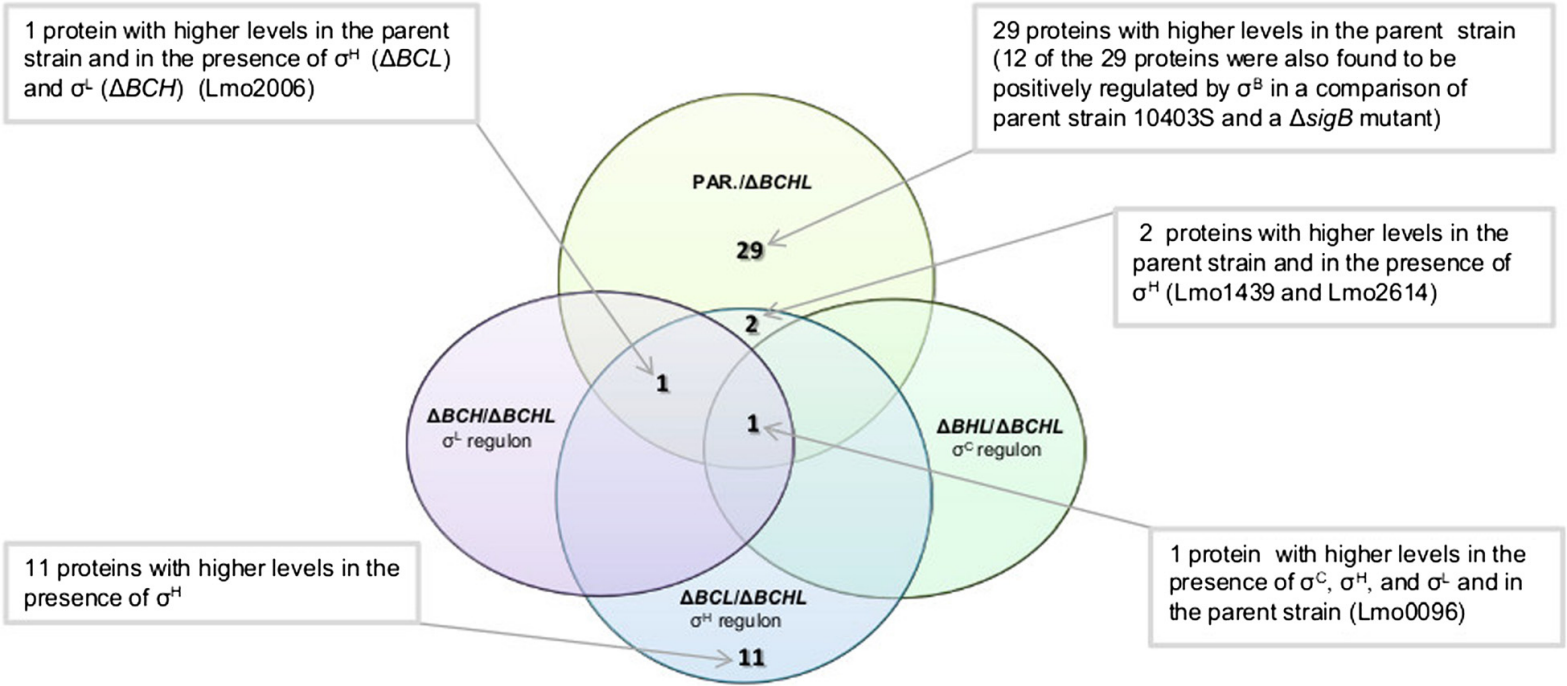
**Venn diagram of proteins identified as showing higher protein levels in comparisons of (i) *L. monocytogenes *parent strain 10403S (PAR.) and Δ *BCHL *; (ii) Δ*BCH *and Δ*BCHL *(identifying genes positively regulated by σ**^**L**^**); Δ*BCL *and Δ*BCHL *(identifying genes positively regulated by σ**^**H**^**); and Δ*BHL *and Δ*BCHL *(identifying genes positively regulated by σ**^**C**^**).** Twelve of the 29 proteins that were found to be positively regulated in the parent strain were also found to be positively regulated by σ^B^ in a recent proteomics study, which compared *L*. *monocytogenes* parent strain 10403S and a Δ*sigB* mutant [23]; these proteins include Lmo2748, Lmo2213, Lmo2158, Lmo2047, Lmo1830, Lmo0913, Lmo0796, Lmo0794, Lmo0722, Lmo0654, Lmo0539, and Lmo0265. The 17 proteins that show higher levels in the parent strain as compared to the Δ*BCHL* strain, but were not identified as positively regulated by any of the alternative σ factors include Lmo1540, Lmo2610, Lmo1422, Lmo1421, Lmo1602, Lmo1426, Lmo1428, Lmo2205, Lmo2398, Lmo1601, Lmo0554, Lmo1634, Lmo0110, Lmo2558, Lmo0783, Lmo0134, and Lmo0098.

Our data reported here also provided an opportunity to gather further insight into genes and proteins that are co-regulated by multiple σ factors and, consequently, into regulatory networks among different alternative σ factors. To facilitate these analyses, we also compared the protein levels between the *L*. *monocytogenes* parent strain and the Δ*BCHL* strain (which does not express any alternative σ factors). This analysis identified (i) 33 proteins that showed significantly higher levels (FC ≥ 1.5; *p*^c^ < 0.05) in the parent strain as compared to the Δ*BCHL* strain (Additional file 1: Table S1) and (ii) 44 proteins that show lower levels in the parent as compared to the Δ*BCHL* mutant (Additional file 1: Table S1). Approximately 40% of the proteins that showed differential production (either up or down) are involved in energy metabolism and transport and binding functions (Figure 1). Among the 33 proteins that showed higher levels in the parent strain, (i) two were also found to be positively regulated by σ^H^; (ii) one was also positively regulated by σ^H^ and σ^L^, and (iii) one was also positively regulated by σ^H^, σ^L^ and σ^C^ (Figure 2; Table 4). In addition, 12 of the 29 proteins that were found to be positively regulated in the parent strain, were also found to be positively regulated by σ^B^ in a recent proteomics study, which compared *L*. *monocytogenes* parent strain 10403S and Δ*sigB* mutant grown to stationary phase under the same conditions as used here [23]. While these 12 proteins likely represent proteins that are positively regulated by σ^B^, the other 17 proteins that showed higher levels in the parent strain as compared to the Δ*BCHL* strain, but were not identified as positively regulated by any of the alternative σ factors, represent candidate proteins for redundant co-regulation by multiple alternative σ factors. Future experiments using an *L*. *monocytogenes* strain that only expresses σ^B^ (i.e., a Δ*CHL* strain) may help to not only further define the σ^B^ regulon, but also allow for further refinement of genes and proteins co-regulated by multiple alternative σ factors. Regulatory redundancy among multiple alternative σ factors has also previously been demonstrated through analyses of *Bacillus subtilis* alternative σ factor mutants; in particular, certain phenotypes displayed by a *B*. *subtilis* triple alternative σ factor deletion mutant were not found among single or double mutants of each of the three alternative σ factors, suggesting regulatory overlaps [29].

**Table 4:** Proteins found to be differentially regulated by at least two of the three alternative sigma factors studied here

	**Regulation by**^**b**^			**Regulation by σ**^**Bc**^	**Differential levels in comparison between parent and Δ*BCHL***
**Protein**^**a**^	**σ**^**H**^	**σ**^**L**^	**σ**^**C**^		
Lmo0027	+	-	NDR	NDR	-
Lmo0096 (MptA)	+	+	+	NDR	+
Lmo0319 (BglA)	+	-	NDR	NDR	-
Lmo1877 (Fhs)	-	-	NDR	NDR	-
Lmo2006 (AlsS)	+	+	NDR	NDR	+
Lmo2094	-	-	-	NDR	-
Lmo2097	-	-	NDR	NDR	-
Lmo2098	-	-	NDR	NDR	NDR

^a^Where available, protein name is shown in parenthesis.

^b^Proteins that were identified here as positively (+) or negatively (−) regulated (absolute FC > 1.5; *p* < 0.05) by a given σ factor are shown; NDR (“not differentially regulated”) indicates that a protein was not found to be differentially regulated between strains with and without a given alternative σ factor.

^c^Data for proteins differentially regulated by σ^B^ were obtained from Mujahid *et al*. [23]; this study compared protein levels between the 10403S parent strain and an isogenic Δ*sigB* strain.

Among the 44 proteins that showed lower levels in the parent strain as compared to the Δ*BCHL* mutant (Additional file 1: Table S1), (i) two also showed evidence for negative regulation by σ^H^ and σ^L^ (Lmo2097 and Lmo1877); (ii) one also showed evidence for negative regulation by σ^H^, σ^L^, and σ^C^ (Lmo2094; located in the same operon as lmo2097). Among these 44 proteins, statistical analyses showed overrepresentation of three role categories, including (i) “energy metabolism” (*p* < 0.01; Odds Ratio = 3.02), (ii) “biosynthesis of cofactors, prosthetic groups, and carriers” (*p* = 0.04; Odds Ratio = 2.72), and (iii) “purines, pyrimidines, nucleosides, and nucleotides” (*p* = 0.04; Odds Ratio = 3.29), as well as underrepresentation of the role category “hypothetical proteins” (*p* = 0.01; Odds Ratio = 0.208).

Overall, our data provide additional evidence that a number of genes and proteins are co-regulated by more than one σ factor. This is consistent with previous microarray studies [7] that have reported considerable overlaps between σ factor regulons in *L*. *monocytogenes*, in particular between the σ^H^ and the σ^B^ regulon. We also identified some proteins with particularly striking patterns of co-regulation, including (i) members of the lmo2093-lmo2099 operon, specifically Lmo2094, which was found to be negatively regulated by σ^H^, σ^L^, and σ^C^ and Lmo2097 and Lmo2098, which were found to be negatively regulated by σ^H^ and σ^L^ (Table 4) and (ii) MptA (Lmo0096), which was found to be positively regulated by σ^H^, σ^L^, and σ^C^ (Table 4). Lmo2094 shows particularly striking negative regulation of protein production by σ^H^, σ^L^, and σ^C^ with respective fold changes of −7.35, -28.99, and −1.82. Although Lmo2094 is annotated as a fuculose-phosphate aldolase, it is part of an operon in which most of the other genes with assigned functions are annotated as being involved in the galactitol degradation pathway. Specifically, the lmo2093 to lmo2099 operon encodes components of a putative PTS galactitol family permease [30], including the PTS system galactitol-specific enzyme IIC (Lmo2096), IIB (Lmo2097), and IIA (Lmo2098) components, as well as a transcription antiterminator (Lmo2099), a tagatose-6-phosphate kinase/1-phosphofructokinase (Lmo2095), an L-fuculose-phosphate aldolase (Lmo2094), and a hypothetical protein (Lmo2093). Therefore, it is possible that Lmo2094 is also involved in this pathway functioning as a tagatose-1,6-biphosphate aldolase. This enzyme converts tagatose-1,6,-biphosphate into glyceraldehyde 3-phosphate and dihydroxyacetone phosphate, which allows both tagatose and galactitol to be used as energy sources for glycolysis [31].

MptA, a component of a permease of the PTS mannose–fructose–sorbose family, which is another one of the seven PTS families of *L*. *monocytogenes*[30], showed the highest fold change in the Δ*BCH* strain as compared to the Δ*BCHL* strain, supporting σ^L^ dependent protein levels (FC = 64.16); fold changes supporting σ^H^ and σ^C^ dependent protein levels were 3.39 and 3.19, respectively. MptA is encoded by a gene that is part of a three-gene operon (*mptACD*[32], which also has been designated as *manLMN*[25]); these three genes encode a mannose-specific PTS system IIAB component, a mannose-specific PTS system IIC component, and a mannose-specific PTS system IID component, respectively [25,32]. Recently, it was suggested that during glucose uptake, MptA dephosphorylates, which directly, or indirectly, inhibits PrfA, the major positive regulator of *L*. *monocytogenes* virulence genes [25]. These findings thus provide for a hypothesis that redundant upregulation of MptA, through multiple alternative σ factors, may provide a critical initial step towards inactivation of PrfA.

## Conclusions

Transcriptional regulation through the interplay between alternative σ factors represents an important component of *L*. *monocytogenes* stress response systems and the ability of this pathogen to regulate gene expression during infection. In addition to transcriptional regulation, alternative σ factors may also regulate protein production post-transcriptionally and/or post-translationally. To allow for further insights into the roles of different alternative σ factors in *L*. *monocytogenes*, we thus completed a global evaluation of alternative σ factor-dependent protein production patterns in *L*. *monocytogenes* stationary phase cells. In concert with previous transcriptomic studies, our data not only provide a further refinement of our understanding of the alternative σ factor regulons in this important pathogen, but also provide clear evidence for co-regulation, by multiple σ factors, of different PTS systems, including one PTS system that has been suggested to be linked to regulation of PrfA. Co-regulation by multiple σ factors can provide sensitive means for fine-tuning of gene expression and protein production under different environmental conditions, as well as redundancy that can ensure gene expression and protein production under different conditions. Consistent with the goals of this study, many of the proteins that were identified as showing production dependent on the presence of alternative σ factors appear to represent indirect regulation by a given σ factor, which will require future confirmation by protein based methods (e.g., Western blots, translational fusions).

## Methods

### Bacterial strains, mutant construction, and growth conditions

Splicing by overlap extension (SOE) PCR and allelic exchange mutagenesis was used to construct Δ*BCL*, Δ*BHL*, Δ*BCH*, and Δ*BCHL* mutant strains in an *L*. *monocytogenes* 10403S background as described previously [13] (Additional file 2: Table S2). All mutations were confirmed by PCR amplification and sequencing of the PCR product. Strains were grown to stationary phase in BHI at 37°C as described previously [33].

### Protein isolation, iTRAQ labeling, and Nano-scale reverse phase chromatography and tandem mass spectrometry (nanoLC-MS/MS)

Protein isolation, digestion, and iTRAQ labeling were performed as previously described [33]. Briefly, proteins were isolated from a 25 ml culture of *L*. *monocytogenes* stationary phase cells. A noninterfering protein assay kit (Calbiochem) and 1D SDS-PAGE were used to verify protein concentration and quality. A total of 100 μg protein of each sample was denatured, reduced, and the cysteine residues were blocked. Protein samples were then digested with sequence-grade-modified trypsin at 37°C for 16 h, and protein digestion efficiency was assessed by SDS-PAGE. Tryptic peptides from *L*. *monocytogenes* parent strain 10403S and Δ*BCL*, Δ*BHL*, Δ*BCH*, and Δ*BCHL* mutant strains were each labeled with iTRAQ reagents, according to the manufacturer’s protocols.

Four labeled protein samples were combined for a single run and fractionated via Isoelectric focusing OffGel electrophoresis (OGE) using an Agilent 3100 OFFGEL Fractionator (Agilent, G3100AA), and subsequent nanoLC-MS/MS was carried out using a LTQ-Orbitrap Velos (Thermo-Fisher Scientific) mass spectrometer as previously described [33]. Two separate biological replicates of the entire proteomics experiment were run for each strain.

### Protein identification and data analysis

All MS and MS/MS raw spectra from iTRAQ experiments were processed using Proteome Discoverer 1.1 for subsequent database search using in-house licensed Mascot Daemon; quantitative processing, protein identification, and data analysis were conducted as previously described [33].

The biological replicates of each experiment were analyzed independently. As described in [33], the Wilcoxon signed rank test was applied to peptide ratios for each identified protein to determine significant changes between strains. The Fisher’s Combined Probability Test was then used to combine FDR adjusted Wilcoxon *p*-values from each replicate into one test statistic for every protein to obtain a combined *p*-value (*p*-value^c^). Proteins with peptide ratios exhibiting a Fisher’s Combined Probability Test *p*-value^c^ < 0.05 and an iTRAQ protein ratio ≥ 1.5 in both replicates were considered significantly differentially expressed. Statistical analyses were conducted using R statistical software.

A Monte Carlo simulation of Fisher’s exact test was used to determine whether the distribution of role categories among proteins identified as differentially regulated by a given σ factor was different from the role category distribution that would be expected by chance (based on the role category primary annotation for all *L*. *monocytogenes* EGD-e genes [26]). Individual Fisher’s exact tests were then used to determine whether individual role categories were over- or under- represented; uncorrected *p*-values were reported, allowing readers to apply corrections if deemed appropriate. Analyses were performed using all role categories assigned to a given gene in the JCVI-CMR *L*. *monocytogenes* EGD-e database. Analyses were only performed for regulons that contained 10 or more proteins (i.e., proteins positively regulated by σ^H^; proteins negatively regulated by σ^L^; proteins with higher or lower levels in the parent strain).

## Competing interests

The authors declare that they have no competing interests.

## Authors’ contributions

SM performed the experimental work and most of the data analysis that was not carried out at the Cornell Proteomics and Mass Spectrometry Core Facility and drafted the manuscript. RHO contributed to analysis of the data and helped to revise the manuscript. MW and KJB conceived of the study, and participated in its design and coordination and helped to draft or revise the manuscript. All authors read and approved the final manuscript.

## Supplementary Material

Additional file 1: Table S1 Proteins found to be differentially produced between *L*. *monocytogenes* parent strain 10403S and Δ*BCHL*. Click here for file

Additional file 2: Table S2 Strains used in this study. Click here for file
